# Targeting Cancer Stem Cells to Overcome Therapy Resistance in Ovarian Cancer

**DOI:** 10.3390/cells9061402

**Published:** 2020-06-04

**Authors:** Sandra Muñoz-Galván, Amancio Carnero

**Affiliations:** 1 Instituto de Biomedicina de Sevilla, IBIS, Hospital Universitario Virgen del Rocío, Universidad de Sevilla, Consejo Superior de Investigaciones Científicas, Avda. Manuel Siurot s/n, 41013 Seville, Spain; 2 CIBERONC, Instituto de Salud Carlos III, 28029 Madrid, Spain

**Keywords:** ovarian cancer, cancer stem cells, therapy resistance

## Abstract

Ovarian cancer is the most lethal gynecological malignancy due to its late detection and high recurrence rate. Resistance to conventional platinum-based therapies and metastasis are attributed to a population of cells within tumors called cancer stem cells, which possess stem-like features and are able to recapitulate new tumors. Recent studies have deepened the understanding of the biology of ovarian cancer stem cells and their special properties and have identified multiple markers and signaling pathways responsible for their self-renewal abilities. Targeting cancer stem cells represents the most promising strategy for overcoming therapy resistance and reducing mortality in ovarian cancer, but further efforts must be made to improve our understanding of the mechanisms involved in therapy resistance. In this review, we summarize our current knowledge about ovarian cancer stem cells, their involvement in metastasis and their interactions with the tumor microenvironment; we also discuss the therapeutic approaches that are being developed to target them to prevent tumor relapse.

## 1. Introduction

Ovarian cancer (OC) is the leading cause of gynecological cancer-related death. Despite the fact that its frequency is lower than that of uterine cancer, it is responsible for 4.4% of cancer-related deaths worldwide, with almost 240,000 new cases per year in the world, and the mortality rate of ovarian cancer is predicted to rise significantly by 2040 [1]. The 5-year overall survival of OC is 45%, but it is as low as 25% in women with advanced-stage disease. This is because most patients (approximately 75%) are diagnosed in advanced stages due to nonspecific clinical manifestations, leading to high mortality rates. In fact, ovarian cancer is known as the silent killer cancer [2]. Complete cytoreductive surgery, achieving resection of all macroscopically visible disease, combined with chemotherapy is the standard treatment for OC. Standard chemotherapy is based on the combination of cisplatin, or its analog carboplatin, with paclitaxel [3], doxorubicin [4,5] or docetaxel [6]. However, 80% of patients in advanced stages and 20% in early stages will eventually relapse, making OC an incurable disease in 50%–60% of cases.

The current tumorigenesis hypothesis proposes that only a small proportion of cancer cells are able to propagate into a tumor. These cells are termed tumor-initiating cells or cancer stem cells (CSCs) and possess pluripotency properties similar to normal stem cells [7,8,9,10]. Recent studies suggest that CSCs remain as a distinct cell population inside tumors that causes relapse and metastasis, leading to the formation of new tumors and chemoresistance [11,12,13]. CSCs are also present in ovarian tumors, where they were detected fifteen years ago and were shown to cause resistance to chemotherapy [14,15,16]. Therefore, the development of new therapies targeting CSCs aims to improve the life of cancer patients, especially those suffering metastatic disease and to avoid relapse in chemotherapy-resistant tumors.

In this review, we discuss our current knowledge about the biology of ovarian cancer stem cells (OCSCs), their involvement in tumorigenesis and metastasis, the influence of the tumor microenvironment on their maintenance and the signaling pathways involved, with a special emphasis on the development of new therapeutic alternatives targeting OCSCs. This strategy is expected to potentially eliminate chemotherapy resistance and prevent tumor relapse, leading to longer survival and a better quality of life for patients.

## 2. Origin and Molecular Features of OC

Traditionally, it was thought that the ovarian surface epithelium (OSE) was the only site of origin of OCs [17]. However, in 2001, preneoplastic lesions were discovered in the fallopian tubes (FT) of high-grade serum ovarian carcinoma (HGSOC) patients [18]. Since then, research has shown that the OSE origin is restricted to some types of OC, while most of them arise from non-ovarian tissues [19]. In the case of the highly abundant HGSOC type, several studies have revealed that patients show early lesions in the FTs called serous tubal intraepithelial carcinomas (STICs), which possess molecular features similar to those of the main lesion, including *TP53* mutations and increased DNA repair and cell cycle gene expression [20,21,22]. Indeed, it has been demonstrated that the precursor lesions of HGSOC are located in the FT epithelium and are driven by *TP53* mutations [23], although in some cases, STICs can also represent metastatic lesions [24]. These observations support a change in our vision of the OC origin.

At the histological level, OC is a heterogeneous disease; epithelial OC is the most common, accounting for approximately 90% of cases. It comprises five main described subtypes: HGSOC, low-grade serous ovarian carcinoma (LGSOC), endometrioid, clear cell and mucinous. In recent years, evidence has shown that each type has unique molecular features, treatment response and prognosis [25]. In contrast, the current classification combines molecular genetics and clinical features and describes two major types of ovarian cancer: type I includes LGSOC, endometrioid, clear cell and mucinous OCs, while type II comprises HGSOC, the principal component, and nonepithelial OC [26,27]. Type I tumors are characterized by a low grade, slow growth rate and being restricted to the ovary at diagnosis, as well as being genetically more stable. In contrast, type II tumors show a high grade, high proliferating rate, and dissemination to the peritoneum or to the omentum, as well as high rates of genomic instability.

In recent years, several studies have shown that both groups of OCs are genetically independent. Type I tumors are characterized by an active mitogen-activated protein kinase (MAPK) pathway, frequently with activating mutations in *KRAS* and *BRAF* but also in *PI*3*K*, *PTEN*, *ARID*1*A*, *Wnt/β*-*catenin* and *ERRB*2 [28]. Type II tumors, which are HGSOC in 75% of cases and are responsible for 80% of deaths related to ovarian cancer, show a common signature characterized by mutations in *TP*53, either activating or inactivating, as well as frequent *BRCA*1 and *BRCA*2 mutations [29]. Overexpression of proto-oncogenes, such as *AKT* and *ERRB*2, also occurs in some cases. They show high genetic instability and increased activity of DNA repair machinery, such as overexpression of poly(ADP-ribose) polymerase (*PARP*). In addition to genetic mutations, epigenetic alterations have also been described in OC, comprising both global DNA hypomethylation and gene-specific hypermethylation. While passive DNA hypomethylation associated with genetic instability and poor prognosis has been detected as blocks in late-replicating and repressed regions [30], specific hypermethylation of CpG sites at gene promoters affects specific tumor suppressor genes such as *SLIT*2, *PTEN*, *OPCML*, *RASSF*1*A*, *p*16, *MLH*1, E-cadherin or *APC* [31].

Regarding prognosis, type I tumors are detected mostly in early stages (I/II) and have a 5-year survival rate of more than 80% after chemotherapy [32]. However, the detection of type II tumors frequently occurs in advanced stages, leading to a poor prognosis. Although their initial response to chemotherapy is promising, death due to disease relapse and therapy resistance of type II tumors occurs in 90% of cases [29]. The development of chemoresistance is thought to be due to persistence or enrichment of OCSCs after treatment [33]; the detection and features of OCSCs are discussed in the next section.

## 3. Biology and Features of Ovarian Cancer Stem Cells

In 1994, a study by Lepidot et al. found that a rare population of CD34+ CD38− acute myeloid leukemia cells was able to establish leukemia after transplantation to the bone marrow of SCID mice [10]. This was the first report of tumor-initiating cells, commonly referred to as CSCs. Since then, increasing evidence has led to the proposal of the CSC hypothesis, according to which a subpopulation of cells within tumors would be responsible for sustaining tumor growth and would be able to generate a new tumor [7,8]. CSCs share features with standard stem cells, including self-renewal and multilineage differentiation capacities, resistance to drugs and stress, quiescence, similar markers and regulation by similar signaling pathways. However, these properties, which are highly regulated in stem cells, are more plastic in CSCs [34].

Ovarian CSCs were identified fifteen years ago by Bapat et al. from the ascites of a patient with advanced OC [14]. The identified transformed clones were able to grow in low attachment conditions and establish tumors in serial transplantations over animal models. OCSCs also possess other stemness properties, such as chemoresistance, increased expression of stem-related genes (such as *OCT*4, *NANOG* or *NESTIN*) and elevated levels of stem cell surface markers (such as CD44 and CD117) [16,35,36]. Stem cells have been identified in the coelomic epithelium of mouse ovaries [37] but also in a transitional region between the ovarian surface epithelium, the mesothelium and the tubal epithelium, known as the hilum [38]. These cells express stem markers, such as ALDH1, LGR5, LEF1, CD133 and CK6B, show increased sphere formation and increased transformation potential upon inactivation of *Trp*53 and *Rb*1, two tumor-suppressor genes frequently mutated in HGSOC. In addition, side population cells with stemness features have been identified in mouse OC cells [39]. These cells overexpress membrane ABC transporters that facilitate the efflux chemotherapeutic agents, leading to therapy resistance [15,40].

The current view that CSCs are responsible for chemotherapy resistance and tumor metastasis opens a broad spectrum of possibilities for targeting residual disease and preventing relapse. To develop this strategy, in particular for the highly resistant OC, reliable detection and molecular characterization of OCSCs is essential; with this information, OCSCs could be used as therapeutic targets. Their identification has commonly been based on their ability to reproduce tumors after transplantation, as well as their increased sphere formation capacity. Additionally, both surface and intracellular markers have been identified, including CD44, CD117, CD24 and CD133 or ALDH1, OCT4, SOX2 and NANOG [36]. However, the use of markers for OCSC identification is still under debate, and there is no consensus yet about universal OCSC markers.

### 3.1. Surface Markers Defining OCSCs

#### 3.1.1. CD44

In the initial identification of OCSCs, it was shown that the hyaluronic acid (HA) receptor CD44 was upregulated [14]. CD44 binding of its ligand leads to interaction with Nanog and pluripotency gene expression in both breast and ovarian cancer cells [41]. In addition, the interaction of Nanog with STAT3 leads to increased expression of *MDR*1 and increased efflux of chemotherapeutic drugs, which results in chemoresistance. In HGSOC, CD44+ CD117+ cells (see below) demonstrated a highly elevated capacity to recapitulate the original tumor in vivo [16]. In addition, OCSCs were identified among epithelial OC cells from ascites and tumors to be CD44+ and MyD88+. These cells showed increased cytokine/chemokine production, high repair capacity and increased formation of spheroids in suspension, as well as the ability to recapitulate the original tumor in vivo and chemoresistance [42]. However, the ability of CD44 to function as a prognostic factor for patient survival remains controversial [43].

#### 3.1.2. CD117 (c-Kit)

As mentioned above, CD117 has been used in combination with CD44 to isolate OCSCs [16]. CD117, also known as c-Kit, is a tyrosine kinase receptor responsible for the activation of several signaling pathways involved in survival, proliferation and migration, and it has been related to tumor progression and stemness [44]. CD117+ cells isolated from OC primary tumors or ascites were able to establish the original tumor with self-renewal and differentiation capacities [45]. In addition, CD117 is overexpressed in OCSCs and mediates both tumor-initiating capacity and chemoresistance to cisplatin/paclitaxel by activating the Wnt/β-catenin-ABCG2 axis [46].

#### 3.1.3. CD24

CD24+ cells were isolated from patient ovarian tumor samples with increased self-renewal capacity and chemoresistance [47]. These cells were able to recapitulate tumors in vivo, in contrast to CD24- cells. This was confirmed in a mouse model of ovarian cancer with conditional deletion of *Apc, Pten* and *Trp*53, where CD24+ cells isolated from mouse tumors were able to initiate new tumors through JAK2-STAT3 signaling [48].

#### 3.1.4. CD133

This marker is a glycosylated membrane protein that was originally described as a CSC marker in brain tumors due to the capacity of CD133+ cells to recapitulate the original tumor from as few as 100 cells [49], although its use as a CSC marker is controversial [50]. In OC, CD133+ cells were shown to have similar features, showing increased tumorigenicity and the ability to recapitulate the original tumor [51]. Indeed, platinum-resistant CD133+ OC cells were shown to divide asymmetrically, leading to both CD133+ and CD133− cells, while CD133− cells only produced other CD133− cells [52]. Interestingly, CD133 gene transcription is epigenetically regulated by promoter DNA methylation and histone acetylation. However, in agreement with the observations in other cancers, OCSCs from patient tumors show heterogeneity in their composition of CD133+ and CD133− populations [53].

### 3.2. Intracellular OCSC Markers

#### 3.2.1. ALDH1 Activity

In addition to cell surface markers, intracellular markers have been used to identify OCSCs. Among them, ALDH1 expression and activity are of the most widely used since its discovery as a marker of normal and malignant breast stem cells [54]. The expression of the ALDH1A1 isoform was first shown to be increased in taxane- and platinum-resistant OC cells, as well as in a high percentage of patient samples, and to be inversely correlated with survival [55]. ALDH+ CD133+ OC cells were able to initiate tumors from just 11 cells, and the presence of ALDH+ CD133+ cells in patient samples was also correlated with reduced survival [56]. The ALDH1A1 gene has been shown to be a β-catenin target, and both are overexpressed in OC spheroids; ALDH1A1 inhibition by a small molecule inhibits OC spheroid formation and cell viability [57]. ALDH expression and activity are also regulated by NF-κB signaling through the RelB-dependent alternative pathway [58]. Despite this evidence, the mechanism by which ALDH enzymes contribute to the CSC phenotype is not known, but some evidence points towards a protective role against DNA damage and increased drug efflux transporters [59,60].

#### 3.2.2. Pluripotency Factors

The key feature of CSCs is their capacity for self-renewal and asymmetric cell division, similar to normal stem cells. Pluripotency transcription factors (TFs), such as Nanog, Oct4 and Sox2, are known to form an interaction network with essential roles in the maintenance of pluripotency both in embryonic stem cells [61] and during embryonic development [62,63], as well as in the reprogramming of somatic cells to pluripotent stem cells [64]. Consistent with the CSC hypothesis, pluripotency factors are also overexpressed in CSCs [65]. In epithelial OC, Oct4 is expressed together with the RNA-binding protein Lin28, correlating with increased tumor grade and growth [66]. Oct4, Nanog and c-Myc have been shown to be expressed in ascites and tumor tissue from OC patients [67]. Pluripotency factors have been shown to be overexpressed in spheres derived from OC cell lines [68], and Sox2 in particular is required for the maintenance of these spheres representing OCSCs, their tumorigenicity and their capabilities of migration, invasion and therapy resistance [69]. Therefore, pluripotency factors represent bona fide markers of OCSCs essential for their self-renewal and tumorigenic properties.

## 4. Metastasis of Ovarian Cancer

Metastasis requires that some tumor cells undergo epithelial-to-mesenchymal transition (EMT), a molecular process that involves the switching of epithelial cells, characterized by markers such as E-cadherin, to mesenchymal cells, with markers such as vimentin. Although EMT was initially identified in embryonic development, aberrant EMT activation occurs frequently in cancer cells, although this is often an ‘incomplete’ or partial EMT, showing markers of both cell fates [70]. Thus, EMT TFs, including members of the Snail, Twist and Zeb families, have been shown to act cooperatively to drive oncogenic transformation [71]. EMT in cancer is closely related to CSCs, providing a mechanistic basis for the observation that metastases are usually resistant to therapy [72]. The acquisition of a mesenchymal phenotype and of stem cell markers was initially observed in immortalized mammary epithelial cells, which were able to form mammospheres [73]. Furthermore, non-CSCs of human breast cancer can generate CSCs through a dedifferentiation process that involves epigenetic regulation of the EMT TF ZEB1, providing a connection between the EMT and the plastic generation of CSCs from non-CSC populations in tumors [74,75]. In OC, the EMT TFs Snail and Slug have been shown to mediate the acquisition of stemness markers and the inactivation of the p53-mediated apoptosis program [76], thus extending the connection between EMT and CSCs to OC.

**Figure 1:**
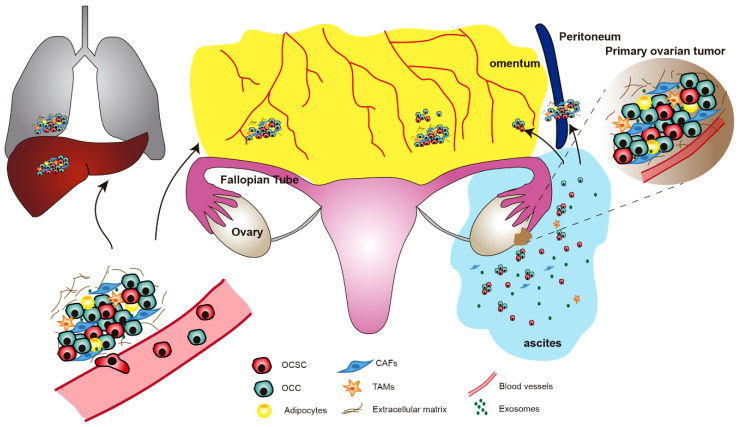
Ovarian Cancer (OC) microenvironment and the implication of Ovarian Cancer Stem Cells (OCSCs) in tumor dissemination. A primary ovarian tumor microenvironment is composed of tumoral cells, non-tumoral cells and the stroma. OCSCs from primary tumors can disseminate through hematogenous metastasis or directly by floating into ascites, which are characterized by a CSC-promoting microenvironment. OCSC, ovarian cancer stem cells; OCC, ovarian cancer cells; CAFs, cancer-associated fibroblasts; TAMs, tumor-associated macrophages.

Metastasis of chemoresistant tumor cells in OC commonly leads to patient death, and OCSCs are essential for this process. Most solid tumors are commonly spread through the hematogenous route, which implies a series of steps, including local invasion, intravasation and survival in blood vessels, extravasation and invasion of new metastatic sites [77]. However, the mechanisms regulating OC metastasis are unique and predominantly different from those of other solid tumors (Figure 1). In this case, the absence of anatomical barriers favors the direct spread of OC tumors to the peritoneal cavity in the form of single cells or spheres composed of tumor cells and some stromal cells, which are able to float in the ascites fluid, leading to multiple metastatic tumors [78]. First, detachment of OC cells and the formation of multicellular aggregates is regulated by the metalloprotease MMP14 [79]. Then, OC spreads preferentially to the peritoneum and, specifically, to the omentum, a peritoneal fold covering the anterior part of the intestine that is rich in adipose tissue [80]. Peritoneal ascites are a rich source of OCSCs [81], providing a nonadherent microenvironment in which only mesenchymal cells are able to survive, and these ascites are also rich in soluble factors that promote tumor growth, such as IL-6, IL-8, IL-10, osteoprotegerin and VEGF [82]. Interestingly, adhesion of OC cells to the peritoneal mesothelium has been shown to occur through the OCSC markers CD44 and integrin-β1, which are recognized by the HA receptor in the membrane of mesothelial cells [83,84]. Indeed, mesothelial cells are able to increase the stem-like properties of OC spheres, suggesting a positive feedback loop in which adhesion and stemness promote each other [85].

Although this particular passive mechanism of tumor metastasis seems to be predominant in OC, it has been recently shown that OC cells are also able to spread by hematogenous metastases (Figure 1) [86]. Circulating tumor cells are thought to share features with CSCs [87], and they have been detected in OC patients [88]. In agreement with the ‘seed and soil’ theory, which establishes the preference or tropism of different cancer types to metastasize in particular organs, circulating OC cells show a preference for dissemination to the omentum. In this case, adhesion occurs between the ERBB3 receptor in the membrane of circulating OC cells and neuregulin 1 (NRG1) secreted by omentum adipocytes [86]. In any case, it is clear that the ability of OC cells to generate metastatic lesions is highly influenced by the host microenvironment, which will be discussed in the next section.

## 5. The Tumor Microenvironment of Ovarian Cancer

The tumor microenvironment is composed of stromal cells and soluble factors that are in dynamic communication with cancer cells, supporting their survival and growth, as well as favoring their invasion and metastasis. In the original site of the tumor, microenvironmental factors may favor the dedifferentiation of cancer cells to CSCs [89,90]. Indeed, we have previously shown that colorectal cancer cells overexpressing phospholipase D2 (PLD2) are able to increase their own stemness by a feedback mechanism in which they induce senescence in neighboring fibroblasts, which in turn secrete a battery of cytokines and growth factors that promote cancer cell stemness [91]. In the particular case of OC, little is known about the possible niche of OCSCs in nonmetastatic lesions. One possibility is that they could benefit from the niche of normal ovarian stem cells. In this regard, ovarian stem cells have been identified in the hilum, i.e., the junction region between the OSE, the mesothelium and the FT [38], but the possible stem cell niche in the FTE remains obscure [36]. On the other hand, the metastatic microenvironment is very well known to play a crucial role in allowing OC cell dissemination and establishment in distant tissues (Figure 1) [92]. In this location, different stromal cell types contribute to the invasive phenotype of OC cells through multiple mechanisms. Furthermore, exosomes derived either from stromal cells or from OC cells play an important role in communication within the microenvironment, and the understanding of this role is increasing.

### 5.1. Stromal Cells in the Metastatic Microenvironment

In OC, several studies have shown that the metastatic tumor microenvironment in the omentum is crucial for metastasis. It contains different types of stromal cells, including adipocytes, mesenchymal stem cells (MSCs), fibroblasts and macrophages, which are known to interact with OC cells, promoting their rapid growth [93]. In addition, multiple layers of regulation have been shown to contribute to complex interactions in the tumor microenvironment, such as those involving cytokines/chemokines, cell type, extracellular matrix components and biomechanical properties [94]. The most abundant cell type in the omentum and peritoneum is adipocytes, which can be reprogrammed to cancer-associated adipocytes through reciprocal interactions with OC cells, which confers them with the ability to release lipids, hormones, adipokines and tumor-promoting factors that favor tumor growth and progression to metastasis [93]. In this regard, the direct transference of lipids from primary adipocytes to cocultured OC cells, together with increased lipolysis in adipocytes and fatty acid β-oxidation in tumor cells, suggests that adipocytes may act as an energy source for cancer cells [95]. In addition, high expression of the fatty acid chaperone FABP4, which is expressed during adipocyte differentiation, in OC primary tumors has been associated with increased relapses after surgery; indeed, FABP4 is induced by Notch1 and promotes tumor growth and angiogenesis in ovarian tumor xenografts [96,97]. Finally, an interesting link between adipocytes and cancer cell metabolism is provided by the finding that centrosome kinase salt-inducible kinase 2 (SIK2) is overexpressed in metastatic OC lesions compared to primary tumors, which promotes metastasis and is activated by adipocytes through calcium-dependent autophosphorylation [98]. Altogether, this evidence suggests that the adipocyte-rich metastatic microenvironment in the peritoneum and omentum induces metabolic changes that favor OC cell survival and growth.

Omental adipose tissue also contains MSCs that contribute to generating a protumorigenic microenvironment, since they have been shown to promote cancer cell growth and vascularization in both ovarian and endometrial cancers [99,100]. MSCs are able to engraft into the stroma and promote tumorigenesis and metastasis in different types of tumors, including ovarian, breast and lung cancers. In OC xenografts, a proinflammatory peptide called LL-37 favors the engraftment of MSCs, tumor growth and angiogenesis, providing a way by which these multipotent cells can be recruited to tumors [101]. Moreover, tumor-associated MSCs show a distinct expression pattern compared with control MSCs, and they promote higher tumor growth of OC cells, possibly leading to more CSCs in a process that is dependent on bone morphogenetic protein (BMP) signaling [102].

The principal cell type composing tumor microenvironments, in both primary and metastatic lesions, is cancer-associated fibroblasts (CAFs), which secrete cytokines, chemokines and growth factors that contribute to cancer progression, invasion and metastasis [103]. Regarding OC, there are CAFs in the omentum before metastatic lesions occur, suggesting that they may contribute to the generation of a premetastatic microenvironment that favors OC cell tropism [104]. CAFs in the omentum can be generated either from MSCs or from normal omental fibroblasts in a process that is influenced by OC cells. Thus, OC cells can reprogram normal omental fibroblasts to CAFs through several microRNAs (miRNAs), including miR-13, miR-155 and miR-214, and cause secretion of chemokines such as CCL5 [105]. In addition, both lysophosphatidic acid and exosomes produced by ovarian tumor cells are able to cause the differentiation of MSCs to CAFs through the TGF-β signaling pathway [106,107]. Indeed, TGF-β plays a key role in the interrelationship between OC cells and CAFs. Normal ovarian fibroblasts treated with TGF-β show upregulation of the *VCAN* gene, encoding versican, through SMAD signaling. This activated NF-κB signaling in OC cells, leading to increased expression of the OCSC markers CD44, MMP-9 and HMMR and to enhanced invasion capacity of these cells [108].

Finally, macrophages are known to be abundant in tumor microenvironments and are referred to as tumor-associated macrophages (TAMs) [109]. TAMs release many molecules that act as mediators of inflammation, such as cytokines, chemokines, growth factors and proteolytic enzymes, leading to an immunosuppressive microenvironment that promotes tumor progression and metastasis [110]. Conversely, OC ascites also promote TAM generation through factors such as LIF and IL-6, which induce the differentiation of monocytes to TAMs [111]. Finally, intraperitoneal TAMs have been shown to promote OC cell spheroid formation and transcoelomic OC metastasis through secretion of epithelial growth factor (EGF), which in turn activates VEGF signaling in tumor cells in a feedback loop that increases tumor progression and migration [112].

### 5.2. Exosomes in the Ovarian Cancer Microenvironment

The role played by exosomes in communication within the tumor microenvironment is important. Exosomes, also called nanovesicles, are extracellular vesicles ranging in size from 30 to 100 nm that are generated from the fusion of intracellular multivesicular bodies before their secretion [113]. They contain different types of signaling molecules, including proteins, RNA, miRNAs and lipids that are responsible for performing important biological functions in receptor cells. In recent years, exosomes have emerged as important modulators of communication between cancer and stromal cells in the tumor microenvironment [114]. In OC, tumor cell-derived exosomes have been shown to play different roles in recipient cells, including modulation of the immune response and control of angiogenesis, growth and migration [115]. Andre et al. first observed that abdominal ascites were a source of exosomes containing tumor-rejection antigens able to promote the expansion of specific cytotoxic T cells in different tumor types, including OC [116]. However, tumor-derived exosomes from OC ascites can also suppress T-cell signaling in a reversible process [117,118]. Other immune cells affected by OC exosomes are macrophages and myeloid cells. While miR222-3p-containing exosomes were able to induce the transition of macrophages to TAMs [119], HSP70-containing exosomes interacted with myeloid-derived suppressive cells, activating them and inhibiting the immune response [120]. Interestingly, these cells were decreased by blocking exosomes with the A8 peptide aptamer, which inhibited tumor progression; this finding highlights the possibility of using exosomes as targets for anticancer therapy [120].

In addition to modulating the immune response, exosomes from malignant ascites in OC patients are rich in soluble E-cadherin and promote tumor angiogenesis through interaction with VE-cadherin and activation of β-catenin and NF-κB signaling in endothelial cells [121]. Exosomes are also inducers of cell migration. For instance, the L1 adhesion molecule contained in OC-derived exosomes is cleaved into a soluble form that is highly abundant in malignant ascites and leads to increased cell migration [122]. Furthermore, exosomes may act as mediators of OC cell-mesothelial cell communication, since CD44 has been shown to be transferred through exosomes to mesothelial cells, inducing the expression of factors that favor tumor cell invasion [123]. Therefore, cancer cell-derived exosomes seem to have a prevalent protumorigenic role through a variety of molecular and cellular processes. However, sometimes they may have antitumor effects, as is the case of exosomes from OC cells containing the tumor-suppressive miR-6126, which is able to target integrin β1, an important mediator of metastasis [124].

Recent studies have also shown that the transfer of different molecules through exosomes can cause resistance to antitumor treatments. In this regard, OC cells resistant to cisplatin are able to export this drug in exosomes, while cisplatin-sensitive OC cells cannot; thus, these sensitive cells have a higher number of lysosomal compartments [125]. However, stromal cells can also produce exosomes that contribute to chemoresistance in OC cells. For instance, both cancer-associated adipocytes and CAFs have been shown to release exosomes containing high levels of miR-21, which binds APAF1 in OC cells conferring resistance to paclitaxel [126]. Exosomes from stromal CAFs also confer resistance to cisplatin [127]. In addition, it has been recently shown that OC cells in hypoxic conditions produce more exosomes, something that occurs in a STAT3-dependent way [128]. Hypoxic exosomes from OC cells derived from patient ascites show higher levels of oncoproteins such as STAT3 and FAS and increase the migration and invasion of OC cells and increase cisplatin efflux leading to chemoresistance. Interestingly, these exosomes were also able to reprogram epithelial secretory cells from the FT to a protumorigenic phenotype [128].

## 6. Therapeutic Strategies in OC

### 6.1. Current Treatments

**Figure 2:**
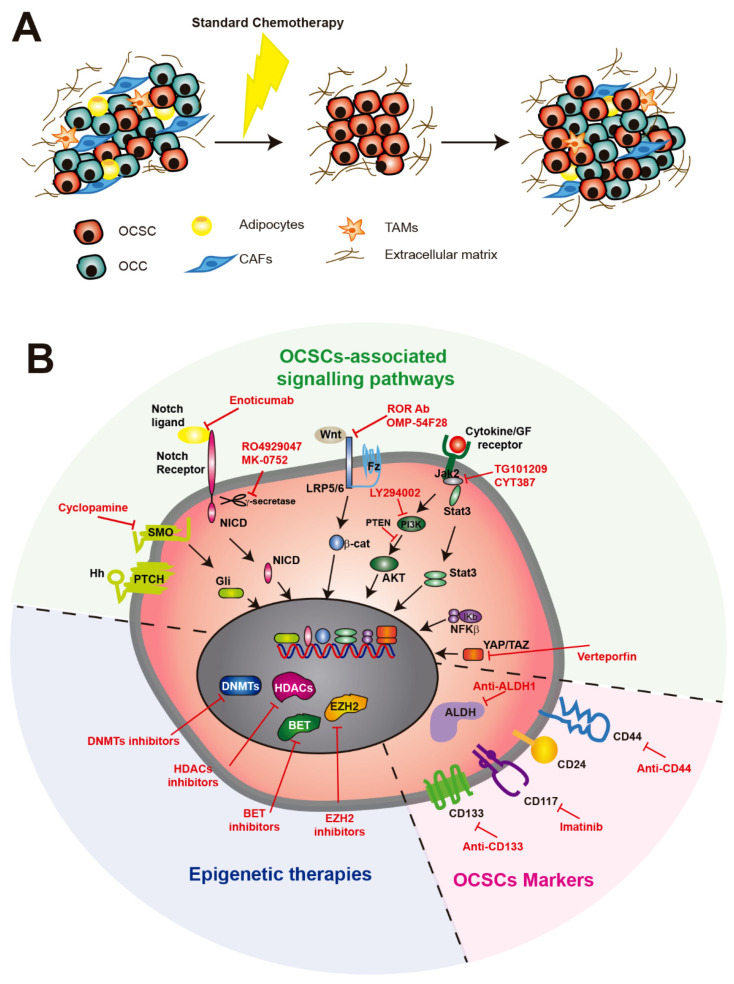
Therapeutic strategies targeting OCSCs. (**A**) Representation of an OC primary tumor before and after standard therapy. Standard chemotherapy removes the bulk tumor but not OCSCs, leading to their enrichment after chemotherapy and consequently to tumor relapse. (**B**) Therapies targeting OCSCs in preclinical or clinical studies, including the targeting of OCSC markers, epigenetic therapies and signaling pathways involved in OCSC generation. OCSC, ovarian cancer stem cells; OCC, ovarian cancer cells; β-cat, β-catenin; Fz, frizzled receptor; Hh, hedgehog; LRP, low-density lipoprotein-related protein; MDR, multidrug resistance; NICD, the intracellular domain of Notch protein; SMO, smoothened; ALDH, aldehyde dehydrogenase; DNMTs, DNA methyltransferases; HDACs, histone deacetylases; BET, bromodomain and extra-terminal; EZH2, histone-lysine N-methyltransferase.

OC is the most lethal gynecological cancer worldwide. Despite its high mortality, the 5-year survival rate of OC patients has not improved much in recent decades, and the standard treatment, consisting of debulking surgery followed by platinum and taxane-based chemotherapy, has remained the same over these years [129]. The overall survival of early-stage disease is greater than 10 years, but most patients are detected in later stages (III or IV) and die within 5 years, which is mainly due to tumor relapse [1]. Although most OC patients respond positively to this treatment, relapse eventually occurs some months later (Figure 2A). Platinum-resistant patients develop recurrence within the next six months following front-line therapy, while platinum-sensitive patients are considered if this takes more than six months. While platinum-sensitive patients are usually treated with double platinum-based chemotherapy, with or without other agents such as doxorubicin, paclitaxel or gemcitabine, different targeted therapies may be used for platinum-resistant patients, including bevacizumab and poly-ADP ribose polymerase (PARP) inhibitors [130].

Patients carrying mutations in *BRCA*1 or *BRCA*2 genes are sensitive to PARP inhibitors, such as olaparib, niraparib and rucaparib. They constitute 50% of HGSOC patients and are also sensitive to platinum-based therapy in most cases, opening the possibility of combining both treatments for these patients [131]. Indeed, several clinical trials with platinum-sensitive patients are obtaining positive results in terms of progression-free survival [132,133,134,135,136]. However, PARP inhibitors have been shown to induce resistance, with an enrichment of CSCs detected as CD133+ and CD117+ cells. This effect is independent of the BRCA mutation status and seems to be due to increased DNA repair in CSCs [137]. The combination of PARP inhibitors with chemo- or radiotherapy may sensitize CSCs to this therapy, as has been shown for glioblastoma and colorectal cancer [138,139].

### 6.2. Targeting OCSCs with Stemness Markers

Provided that CSCs facilitate metastasis and therapy resistance, it is clear that targeting CSCs is likely a promising strategy for overcoming recurrence [140]. In highly resistant cancers such as OC, this becomes crucial, and several therapeutic approaches have been explored to target OCSCs, showing generally positive results alone or in combination with traditional therapies [141]. However, most of these anti-CSC therapies target stemness factors (Figure 2B) that are shared with normal stem cells, compromising their safety. For instance, both monoclonal antibodies targeting CD44 and siRNAs against *CD*44 mRNA attached to a paclitaxel delivery system show increased OCSC death [142,143]. In addition, inhibition of the interaction between CD44 and the HA receptor of mesothelial cells using small oligosaccharides leads to decreased tumorigenesis [144]. However, a previous study using an antibody-drug conjugate bivatuzumab mertansine, which targets CD44v6, led to severe skin-related adverse effects that resulted in one case of fatality related to the drug and the termination of the clinical trial before reaching the maximum tolerated dose [145]. The tyrosine kinase inhibitor imatinib mesylate, which targets CD117, PDGFR-α and PDGFR−β, was also tested in epithelial OC with poor results as a single agent [146]. Finally, two ALDH1A1 inhibitors have been recently described to target OCSCs. The inhibitor 673A preferentially targets CD133+ OC cells and induces necroptosis by promoting mitochondrial uncoupling protein expression and reducing oxidative phosphorylation. Its use in combination with cisplatin causes promising tumor reduction [147]. On the other hand, the small inhibitor molecule CM37 decreases OC proliferation and stemness markers in cell lines and malignant ascites by increasing reactive oxygen species-mediated DNA damage [148]. However, none of these compounds are currently being used in clinical trials.

### 6.3. Epigenetic Therapies Against OCSCs

The acquisition of malignant phenotypes by cancer cells is the result of both intrinsic and extrinsic mechanisms, i.e., mutations and environmental factors, respectively, that modulate their epigenome and determine which cancer cells conserve the self-renewal capacity as CSCs [149]. Since CSC maintenance requires reprogramming of the epigenome, it seems likely that modulating it could cause CSCs to transition to a more differentiated stage, making them sensitive to current treatments. For that reason, several epigenetic drugs have been tested in recent years (Figure 2B). For example, the DNA methyltransferase (DNMT) 1 inhibitor guadecitabine resensitized ALDH1+ OCSCs to cisplatin and showed positive results in a phase I trial [150,151]. Histone deacetylases (HDACs) 1 and 7 were shown to be required for the maintenance of OCSCs, and HDAC7 overexpression was enough to induce the CSC phenotype [152]. This opened the possibility of using HDAC inhibitors in combination with other drugs to overcome resistance in OC [153]. This is not the case for histone methyltransferase inhibitors, provided that histone-lysine N-methyltransferase EZH2 shows contradictory results regarding OCSCs, probably due to their pleiotropic functions [154]. Finally, bromodomain and extra-terminal (BET) inhibitors have recently shown promising results. Inhibition of BRD4 by the small molecule JQ1 showed antitumor effects in HGSOC patients overexpressing MYC [155]. Furthermore, JQ1 decreased the ALDH1 activity of OCSCs by targeting a super-enhancer bound by BRD4, and its combination with cisplatin improved survival in vivo [156].

### 6.4. Therapies Targeting Signaling Pathways Involved in OCSC Generation

Multiple signaling pathways have been described to play a role in the generation and maintenance of CSCs. Since CSCs are responsible for chemotherapy resistance, these pathways represent promising strategies to specifically target CSCs and overcome recurrence [157]. Treatments based on targeting developmental signaling pathways, including Wnt, hedgehog (Hh) and Notch, are being tested in clinical trials with variable results [158], probably due to their high crosstalk and pleiotropy. However, other pathways have been involved in CSC in general and OCSC in particular (Figure 2B). We will discuss recent advances in how these pathways work in OC and in the generation of OCSCs, as well as their therapeutic targeting.

#### 6.4.1. Notch Pathway

Notch3 is a recurrent amplified gene in HGSOC that is related to increased proliferation and survival of OC cells and reduced patient survival [159,160]. In addition, the most highly expressed Notch3 ligand in OC cells and mesothelial cells in the peritoneum is Jagged 1 (Jag1), which has been shown to promote OC cell proliferation and adhesion, suggesting a role for the Jag1/Notch3 axis in tumor dissemination [161]. On the other hand, Notch3 overexpression in OC cell lines leads to increased expression of stemness genes, such as pluripotency transcription factors and ABC transporters, suggesting a role in the generation of OCSCs (Park, Am J Pathol 2010). Indeed, Notch3 overexpression in OC cells increases the number of CSCs and resistance to cisplatin [162]. Interestingly, treatment of cells with γ-secretase inhibitor 1 (GSI), a Notch pathway inhibitor, eliminates OCSCs by inducing the DNA damage response, cell cycle arrest and apoptosis, while a combination of GSI and cisplatin leads to full depletion of both OCSCs and bulk tumor cells [162]. Although the γ-secretase inhibitor RO4929097 did not show enough activity as a single agent in a phase II clinical trial [163], another inhibitor, MK-0752, led to apoptosis and inhibition of tumor growth in xenograft models when used sequentially after cisplatin treatment [164]. Finally, a phase I trial using enoticumab, a monoclonal antibody that targets delta-like (Dll) 4 and disrupts Notch signaling, has shown a good response and toleration in OC patients [165]. Altogether, these data suggest that the Notch pathway is a promising candidate for targeting OC tumors in combination with platinum therapy.

#### 6.4.2. Wnt Pathway

This pathway functions in a wide variety of developmental processes involving many different tissues and organs. In the ovary, the Wnt pathway has been implicated in the normal development of stem cells in the OSE and FT, where the G-protein coupled receptor Lgr5 is expressed [38,166,167]. The Wnt pathway is frequently involved in cancer development and is a candidate for therapy targeting CSCs [168]. In OC, OCSCs express the tyrosine kinase-like receptor ROR1, which recognizes the Wnt5 ligand and is required for migration, invasion, spheroid formation and tumor engraftment in nude mice. Treatment with a ROR1-specific monoclonal antibody inhibits these phenotypes, avoiding xenograft growth and depleting CSCs [169]. On the other hand, the OCSC marker c-KIT is highly expressed in hypoxic tumor microenvironments, which leads to β-catenin activation and high expression of ABC transporters that cause resistance to cisplatin/paclitaxel [46]. Interestingly, inhibition of c-KIT with imatinib in combination with cisplatin/paclitaxel suppressed tumor growth in OC cells and in vivo models. Finally, a novel Wnt pathway inhibitor that is currently being tested is ipafricept (OMP-54F28), a fusion protein comprising the extracellular part of frizzled receptor 8 (Fzd8) fused to an IgG1 Fc fragment that competes with frizzled receptors for binding Wnt ligands [170]. This chimeric protein was previously tested in preclinical models showing high efficacy against tumor growth and CSCs, alone or in combination with other agents [171].

#### 6.4.3. Hedgehog Pathway

Several members of the Hh pathway, including the Hh receptor Patched and the Gli1 TF, are overexpressed in OC patients; patients with overexpression of these molecules have shorter survival than those without overexpression [172]; in addition, inhibition of the Hh pathway with cyclopamine, which interferes with smoothened signal transduction, blocks tumor growth both in vitro and in vivo [173]. Activation of the Hh pathway is also related to OCSCs, since treatment with Hh ligands (Shh and Ihh) leads to more sphere-forming cells, whereas cyclopamine treatment has the opposite effect [174]. However, the Hh pathway is also related to chemoresistance, as cisplatin-resistant OC cells treated with Gli1 shRNA show decreased DNA repair of cisplatin-induced DNA adducts [175]. Further studies will be necessary to assess whether inhibition of the Hh pathway effectively results in selective elimination of OCSCs and prevents tumor recurrence.

#### 6.4.4. Hippo Pathway

It is known that this pathway is involved in OC development; in particular, its effector protein YAP is encoded by a well-known oncogene in OC [176,177]. Alterations in the Hippo pathway may be in the origin of OCs. For instance, *YAP* is highly expressed in cancerous FT tissue, and its overexpression in immortalized FT secretory epithelial cells leads to increased proliferation and tumor formation [178]. Additionally, YAP hyperactivation leads to senescence in OSE cells, while the loss of its regulator LATS2 switches these cells from a senescence phenotype to a malignant transformation phenotype [179]. However, YAP and its coactivators, the TEAD TFs, have also been described to promote OCSC formation from tumor xenografts [180]. In this regard, we recently showed that myosin phosphatase target subunit 1 (*MYPT*1), a regulatory subunit of protein phosphatase 1 (PP1), is downregulated in OC patients, leading to decreased survival and that its downregulation in OC cell lines and xenografts, either with shRNA or with overexpression of *miR*-30*b*, leads to increased tumorigenesis and platinum therapy resistance [68]. We provide evidence that *MYPT*1 downregulation results in increased stemness properties, such as pluripotency factor and surface marker overexpression or sphere formation and that this is mediated by the activation of YAP-dependent gene expression. Finally, YAP inhibition using verteporfin suppresses platinum resistance both in vitro and in vivo, suggesting that targeting the Hippo pathway could be an effective strategy to prevent recurrence in patients with low levels of *MYPT1* [68].

#### 6.4.5. PI3K/PTEN/AKT Signaling

Activation of AKT signaling, indicated by AKT phosphorylation, has been detected in HGSOC tumors. In addition, the use of the PI3K inhibitor LY294002 was shown to increase platinum-mediated apoptosis specifically in OC cell lines with high levels of pAKT, suggesting its possible use as a therapeutic alternative for patients with an active AKT pathway [181]. This pathway is activated in OCSCs since spheroids from OC cell lines have high pAKT levels and low *PTEN* expression. Interestingly, treatment with LY294002 reduced the stemness of spheroid cells and sensitized them to paclitaxel [182]. *AKT*1 depletion with siRNAs decreased spheroid formation and migration, as well as stem marker expression and paclitaxel resistance [183]. Altogether, these data suggest that PI3K/PTEN/AKT is important for CSC formation, maintenance and chemoresistance to paclitaxel, and it is, therefore, a possible therapeutic target for OCSCs [184].

#### 6.4.6. Jak2/STAT3 Signaling

This pathway is activated in most OCs. Activation of STAT3 by phosphorylation of Tyr705 and inactivation of its inhibitor PIAS3 resulted in the initiation of lesions in the FT preceding HGSOC [185]. However, the Jak2/STAT3 pathway is also involved in OCSCs, since CD24+ cells from primary tumors in an apc- pten- trp53- mouse model showed increased pSTAT3 and NANOG expression. Treatment of mice injected with CD24+ cells with cisplatin and the Jak2 inhibitor TG101209 led to increased survival and suppression of metastases [48]. Finally, tumor cells from patient ascites and OC cell lines treated with paclitaxel showed increased levels of CSC markers, with activation of the Jak2/STAT3 pathway, suggesting that it regulates stemness in OCSCs [186]. Treatment of cells with paclitaxel and another Jak2 inhibitor, CYT387, led to pathway inhibition and decreased stem marker levels and sensitivity to treatment. These studies suggest that Jak2 inhibitors may be used together with other common treatments in OC to reduce the pool of OCSCs.

#### 6.4.7. NF-κB Pathway

This pathway has been suggested to play a role in CSC generation [187], and in OC, CD44+ CSCs were shown to have a constitutively active NF-κB pathway [42]. A recent report using a mouse xenograft model of OC showed that this pathway affects OC cells in different ways [58]. While the canonical NF-κB pathway through the RelA TF was essential for the proliferation of OC cells, the alternative pathway mediated by RelB was not and instead affected *ALDH1A*2, *CD*44 and *NANOG* expression in ALDH+ OCSCs. RelB knockdown with shRNA restored chemosensitivity to carboplatin in these cells [58], suggesting that the nonclassical NF-κB pathway could be an interesting target to complement platinum-based chemotherapy and eliminate resistant OCSC populations.

## 7. Conclusions and Perspectives

In recent years, an increasing number of reports have provided strong evidence that tumors are heterogeneous in terms of cell composition and that the CSC subpopulation plays key roles in tumorigenesis, migration, invasion and chemoresistance. Therefore, important efforts are being made to understand the biology and special features of these stem-like cells. This is especially important for developing new therapeutic strategies that overcome resistance to conventional treatments and improve the survival of cancer patients. In cancers such as OC that are typically detected in late stages and have high recurrence rates, finding markers in these CSCs that could be targeted by new therapies is the most promising strategy for decreasing mortality. However, CSC populations are highly heterogeneous and dynamic and are influenced by interactions with the tumor microenvironment [188]. This challenges the possibility of identifying a single treatment targeting CSCs and, instead, supports the idea that the efficacy of different strategies depends on the genetic and epigenetic features of specific CSC populations both within the same patient and among different patients. In this regard, we recently showed that in a cohort of OC patients treated with platinum, the expression of different candidate predictive biomarkers was heterogeneous among platinum-resistant patients, but all of them show increased OCSC marker expression [159]. Indeed, treatment of OC cell lines with platinum combined with inhibitors of different pathways, including Notch, c-Kit, MAPKs and PI3K, led to a reduced number of spheres in all cases, suggesting that many of the identified OCSC-related pathways may also play a role in chemoresistance. Therefore, the identification of markers able to predict the sensitivity to different OCSC pathway inhibitors is key for the future stratification of patients. This would contribute to the ability of personalized medicine strategies to determine the best treatment for each patient, among a battery of available therapies, based on the specific genetic and epigenetic features of each tumor.
